# *N*-(4-Sulfamoylphen­yl)acetamide

**DOI:** 10.1107/S1600536812011701

**Published:** 2012-03-24

**Authors:** Abdullah M. Asiri, Hassan M. Faidallah, Tariq R. Sobahi, Seik Weng Ng, Edward R. T. Tiekink

**Affiliations:** a Chemistry Department, Faculty of Science, King Abdulaziz University, PO Box 80203, Jeddah, Saudi Arabia; b The Center of Excellence for Advanced Materials Research, King Abdulaziz University, Jeddah, PO Box 80203, Saudi Arabia; c Department of Chemistry, University of Malaya, 50603 Kuala Lumpur, Malaysia

## Abstract

In the title compound, C_8_H_10_N_2_O_3_S, the dihedral angle between the acetamide group and the benzene ring is 15.59 (12)° and the amino group is close to being perpendicular to the benzene ring [N—S—C_ar_—C_ar_ (ar = aromatic) torsion angle = 109.4 (2)°]. In the crystal, mol­ecules are linked into supra­molecular tubes parallel to [001] by amine–amide N—H⋯O inter­actions and these are connected into the three-dimensional architecture by amide–sulfonamide N—H⋯O hydrogen bonds. The crystal studied was a racemic twin.

## Related literature

For background to the biological applications of related sulfonamides, see: Croitoru *et al.* (2004 ▶); Dogruer *et al.* (2010 ▶). For related structures, see: Asiri *et al.* (2011 ▶, 2012 ▶).
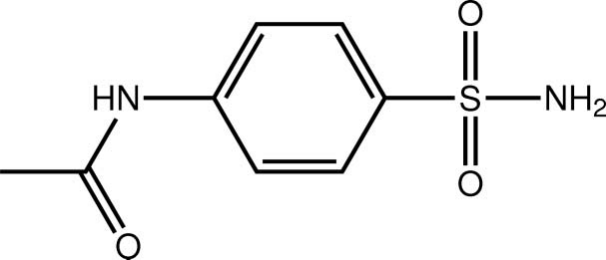


## Experimental

### 

#### Crystal data

C_8_H_10_N_2_O_3_S
*M* *_r_* = 214.24
Tetragonal, 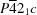

*a* = 15.2631 (4) Å
*c* = 8.0571 (4) Å
*V* = 1877.00 (11) Å^3^
*Z* = 8
Mo *K*α radiation
μ = 0.33 mm^−1^
*T* = 100 K
0.40 × 0.05 × 0.05 mm

#### Data collection

Agilent SuperNova Dual diffractometer with an Atlas detector
Absorption correction: multi-scan (*CrysAlis PRO*; Agilent, 2011 ▶) *T*_min_ = 0.880, *T*_max_ = 0.984
3827 measured reflections
1862 independent reflections
1698 reflections with *I* > 2σ(*I*)
*R*_int_ = 0.028

#### Refinement

*R*[*F* ^2^ > 2σ(*F* ^2^)] = 0.032
*wR*(*F* ^2^) = 0.079
*S* = 1.02
1862 reflections
140 parameters
3 restraints
H atoms treated by a mixture of independent and constrained refinement
Δρ_max_ = 0.25 e Å^−3^
Δρ_min_ = −0.27 e Å^−3^
Absolute structure: Flack (1983 ▶), 625 Friedel pairs
Flack parameter: 0.48 (9)

### 

Data collection: *CrysAlis PRO* (Agilent, 2011 ▶); cell refinement: *CrysAlis PRO*; data reduction: *CrysAlis PRO*; program(s) used to solve structure: *SHELXS97* (Sheldrick, 2008 ▶); program(s) used to refine structure: *SHELXL97* (Sheldrick, 2008 ▶); molecular graphics: *ORTEP-3* (Farrugia, 1997 ▶) and *DIAMOND* (Brandenburg, 2006 ▶); software used to prepare material for publication: *publCIF* (Westrip, 2010 ▶).

## Supplementary Material

Crystal structure: contains datablock(s) global, I. DOI: 10.1107/S1600536812011701/hb6682sup1.cif

Structure factors: contains datablock(s) I. DOI: 10.1107/S1600536812011701/hb6682Isup2.hkl

Supplementary material file. DOI: 10.1107/S1600536812011701/hb6682Isup3.cml

Additional supplementary materials:  crystallographic information; 3D view; checkCIF report

## Figures and Tables

**Table 1:** Hydrogen-bond geometry (Å, °)

*D*—H⋯*A*	*D*—H	H⋯*A*	*D*⋯*A*	*D*—H⋯*A*
N1—H1⋯O3^i^	0.88 (1)	2.08 (1)	2.935 (3)	163 (3)
N1—H2⋯O3^ii^	0.89 (1)	2.04 (1)	2.929 (3)	178 (3)
N2—H3⋯O1^iii^	0.88 (1)	2.34 (2)	3.156 (3)	155 (2)

Symmetry codes: (i) 
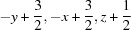
; (ii) 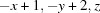; (iii) 
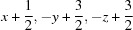
.

**Fig. 1.**
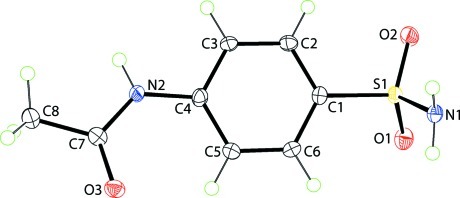
The molecular structure of (I) showing displacement ellipsoids at the 50% probability level.

**Fig. 2.**
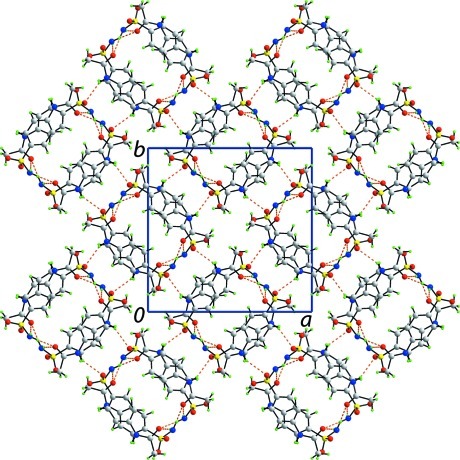
A view in projection down the c axis of the unit-cell contents of (I). The N—H···O hydrogen bonds are shown as orange dashed lines.

## supplementary crystallographic information

### Comment

The crystal and molecular structure of *N*-(4-sulfamoylphenyl)acetamide (I) is reported herein in continuation of on-going structural studies of sulfonamide derivatives (Asiri *et al.*, 2011; Asiri *et al.*, 2012), of interest owing to their biological activity, for example, to selectively inhibit COX–2 (Croitoru *et al.*, 2004) and as they exhibit anti-microbial and anti-fungal activities (Dogruer *et al.* 2010).

In (I), Fig. 1. the amide residue is twisted out of the plane of the benzene ring to which it is attached as seen in the value of the C7—N2—C4—C3 torsion angle of -166.2 (2)°, and the amino group occupies a position perpendicular to the benzene ring with the N1—S1—C1—C2 torsion angle being 109.4 (2)°.

Each of the N—H hydrogen atoms forms a hydrogen bond to an oxygen atom with the amide-O3 atom being bifurcated, Table 1. The amino-H atoms bridge the amide-O atoms to generate supramolecular tubes along the *c* axis. These are connected into the three-dimensional architecture by amide-*H*···*O*(sulfonamide) hydrogen bonds, Fig. 2 and Table 1.

### Experimental

2-Acetyl chloride (0.784 g, 25 mmol) in pyridine (5 ml) was slowly added to a solution of sulfanilamide (2.00 g, 11 mmol) in pyridine (20 ml) and the reaction mixture was stirred at 258 K for 4 h under anhydrous conditions. After warming the solution to room temperature, the pyridine was removed *in vacuo* and the resulting white solid dissolved in ethyl acetate. The organic extract was washed with 3 *M* hydrochloric acid (30 ml) then with saturated sodium bicarbonate solution (30 ml) and finally with brine. Drying over magnesium sulfate and evaporation yielded a white solid which was recrystallized from ethanol to give the title compound as colourless prisms. Yield: 74%. *M*.pt: 491–492 K.

### Refinement

Carbon-bound H-atoms were placed in calculated positions [C—H = 0.95 to 0.98 Å, *U*_iso_(H) = 1.2 to 1.5*U*_eq_(C)] and were included in the refinement in the riding model approximation. The N—H atoms were located in a difference Fourier map, and were refined with a distance restraint of N—H = 0.88±0.01 Å; their *U*_iso_ values were refined. Owing to poor agreement, the (7 7 0) reflection was omitted from the final cycles of refinement. The Flack (Flack, 1983) parameter was calculated from 625 Friedel pairs. The refined value, *i.e*. 0.48 (9). indicates that the crystal examined was a racemic twin.

### Crystal data

**Table:**

C_8_H_10_N_2_O_3_S	*D*_x_ = 1.516 Mg m^−^^3^
*M**_r_* = 214.24	Mo *K*α radiation, λ = 0.71073 Å
Tetragonal, *P*42_1_*c*	Cell parameters from 2081 reflections
Hall symbol: P -4 2n	θ = 2.7–27.5°
*a* = 15.2631 (4) Å	µ = 0.33 mm^−^^1^
*c* = 8.0571 (4) Å	*T* = 100 K
*V* = 1877.00 (11) Å^3^	Prism, colourless
*Z* = 8	0.40 × 0.05 × 0.05 mm
*F*(000) = 896	

### Data collection

**Table:**

Agilent SuperNova Dual diffractometer with an Atlas detector	1862 independent reflections
Radiation source: SuperNova (Mo) X-ray Source	1698 reflections with *I* > 2σ(*I*)
Mirror monochromator	*R*_int_ = 0.028
Detector resolution: 10.4041 pixels mm^-1^	θ_max_ = 27.6°, θ_min_ = 2.7°
ω scan	*h* = −12→19
Absorption correction: multi-scan (*CrysAlis PRO*; Agilent, 2011)	*k* = −18→10
*T*_min_ = 0.880, *T*_max_ = 0.984	*l* = −10→6
3827 measured reflections	

### Refinement

**Table:**

Refinement on *F*^2^	Secondary atom site location: difference Fourier map
Least-squares matrix: full	Hydrogen site location: inferred from neighbouring sites
*R*[*F*^2^ > 2σ(*F*^2^)] = 0.032	H atoms treated by a mixture of independent and constrained refinement
*wR*(*F*^2^) = 0.079	*w* = 1/[σ^2^(*F*_o_^2^) + (0.0379*P*)^2^ + 0.7254*P*] where *P* = (*F*_o_^2^ + 2*F*_c_^2^)/3
*S* = 1.02	(Δ/σ)_max_ = 0.001
1862 reflections	Δρ_max_ = 0.25 e Å^−^^3^
140 parameters	Δρ_min_ = −0.27 e Å^−^^3^
3 restraints	Absolute structure: Flack (1983), 625 Friedel pairs
Primary atom site location: structure-invariant direct methods	Flack parameter: 0.48 (9)

### Fractional atomic coordinates and isotropic or equivalent isotropic displacement parameters (Å^2^)

**Table:**

	*x*	*y*	*z*	*U*_iso_*/*U*_eq_	
S1	0.42050 (3)	0.72593 (3)	0.79689 (8)	0.01459 (14)	
O1	0.38712 (11)	0.72467 (12)	0.6291 (2)	0.0220 (4)	
O2	0.43783 (11)	0.64415 (10)	0.8775 (2)	0.0212 (4)	
O3	0.71401 (10)	1.06596 (10)	0.73297 (19)	0.0184 (4)	
N1	0.34929 (12)	0.77773 (13)	0.9064 (3)	0.0165 (4)	
H1	0.364 (2)	0.783 (2)	1.0119 (16)	0.038 (9)*	
H2	0.329 (2)	0.8243 (14)	0.853 (4)	0.053 (11)*	
N2	0.75181 (12)	0.92922 (13)	0.8186 (2)	0.0165 (4)	
H3	0.7976 (12)	0.8991 (16)	0.852 (3)	0.025 (8)*	
C1	0.51900 (14)	0.78616 (14)	0.7979 (3)	0.0153 (4)	
C2	0.59105 (15)	0.75505 (15)	0.8838 (3)	0.0163 (5)	
H2A	0.5884	0.7002	0.9394	0.020*	
C3	0.66665 (15)	0.80408 (14)	0.8881 (3)	0.0164 (5)	
H3A	0.7165	0.7826	0.9459	0.020*	
C4	0.67072 (14)	0.88484 (15)	0.8086 (3)	0.0155 (5)	
C5	0.59846 (15)	0.91651 (16)	0.7215 (4)	0.0227 (5)	
H5	0.6011	0.9714	0.6662	0.027*	
C6	0.52250 (16)	0.86653 (15)	0.7170 (4)	0.0226 (5)	
H6	0.4727	0.8873	0.6583	0.027*	
C7	0.77054 (15)	1.01386 (14)	0.7821 (3)	0.0158 (4)	
C8	0.86506 (15)	1.03931 (15)	0.8005 (3)	0.0196 (5)	
H8A	0.8689	1.1001	0.8392	0.029*	
H8B	0.8946	1.0339	0.6930	0.029*	
H8C	0.8934	1.0006	0.8813	0.029*	

### Atomic displacement parameters (Å^2^)

**Table:**

	*U*^11^	*U*^22^	*U*^33^	*U*^12^	*U*^13^	*U*^23^
S1	0.0124 (3)	0.0141 (3)	0.0172 (2)	−0.0010 (2)	0.0011 (2)	−0.0006 (3)
O1	0.0201 (8)	0.0260 (9)	0.0199 (8)	−0.0028 (8)	−0.0005 (7)	−0.0042 (8)
O2	0.0197 (9)	0.0116 (8)	0.0323 (9)	−0.0012 (7)	0.0006 (8)	0.0017 (7)
O3	0.0197 (8)	0.0153 (8)	0.0202 (8)	0.0024 (6)	0.0016 (7)	0.0017 (7)
N1	0.0122 (9)	0.0199 (10)	0.0176 (9)	0.0014 (8)	0.0033 (9)	0.0026 (9)
N2	0.0103 (9)	0.0147 (9)	0.0247 (10)	0.0011 (7)	0.0004 (8)	0.0038 (9)
C1	0.0129 (10)	0.0167 (10)	0.0163 (9)	−0.0010 (9)	0.0012 (10)	−0.0009 (11)
C2	0.0174 (11)	0.0119 (10)	0.0197 (11)	0.0028 (9)	0.0001 (10)	0.0017 (9)
C3	0.0138 (11)	0.0140 (11)	0.0215 (11)	0.0039 (9)	−0.0002 (10)	0.0041 (10)
C4	0.0119 (10)	0.0148 (10)	0.0197 (11)	0.0010 (8)	0.0020 (10)	−0.0012 (10)
C5	0.0167 (11)	0.0188 (11)	0.0326 (13)	−0.0003 (9)	−0.0011 (11)	0.0109 (12)
C6	0.0147 (11)	0.0237 (12)	0.0296 (12)	0.0015 (10)	−0.0034 (12)	0.0107 (12)
C7	0.0180 (11)	0.0145 (10)	0.0150 (10)	0.0012 (9)	0.0035 (10)	−0.0010 (10)
C8	0.0188 (11)	0.0186 (11)	0.0214 (11)	−0.0034 (9)	0.0003 (11)	0.0012 (11)

### Geometric parameters (Å, º)

**Table:**

S1—O2	1.4316 (17)	C2—C3	1.376 (3)
S1—O1	1.4446 (17)	C2—H2A	0.9500
S1—N1	1.608 (2)	C3—C4	1.391 (3)
S1—C1	1.762 (2)	C3—H3A	0.9500
O3—C7	1.238 (3)	C4—C5	1.394 (3)
N1—H1	0.880 (10)	C5—C6	1.388 (3)
N1—H2	0.885 (10)	C5—H5	0.9500
N2—C7	1.355 (3)	C6—H6	0.9500
N2—C4	1.413 (3)	C7—C8	1.501 (3)
N2—H3	0.878 (10)	C8—H8A	0.9800
C1—C2	1.384 (3)	C8—H8B	0.9800
C1—C6	1.390 (3)	C8—H8C	0.9800
			
O2—S1—O1	118.55 (11)	C4—C3—H3A	119.7
O2—S1—N1	107.74 (11)	C3—C4—C5	120.3 (2)
O1—S1—N1	106.36 (10)	C3—C4—N2	115.9 (2)
O2—S1—C1	107.16 (10)	C5—C4—N2	123.8 (2)
O1—S1—C1	108.19 (11)	C6—C5—C4	118.9 (2)
N1—S1—C1	108.52 (11)	C6—C5—H5	120.5
S1—N1—H1	114 (2)	C4—C5—H5	120.5
S1—N1—H2	111 (2)	C5—C6—C1	120.3 (2)
H1—N1—H2	119 (3)	C5—C6—H6	119.9
C7—N2—C4	128.99 (19)	C1—C6—H6	119.9
C7—N2—H3	113.4 (18)	O3—C7—N2	122.3 (2)
C4—N2—H3	117.6 (18)	O3—C7—C8	122.33 (19)
C2—C1—C6	120.5 (2)	N2—C7—C8	115.34 (19)
C2—C1—S1	120.10 (17)	C7—C8—H8A	109.5
C6—C1—S1	119.40 (18)	C7—C8—H8B	109.5
C3—C2—C1	119.5 (2)	H8A—C8—H8B	109.5
C3—C2—H2A	120.2	C7—C8—H8C	109.5
C1—C2—H2A	120.2	H8A—C8—H8C	109.5
C2—C3—C4	120.5 (2)	H8B—C8—H8C	109.5
C2—C3—H3A	119.7		
			
O2—S1—C1—C2	−6.7 (2)	C2—C3—C4—N2	−179.1 (2)
O1—S1—C1—C2	−135.60 (19)	C7—N2—C4—C3	−166.2 (2)
N1—S1—C1—C2	109.4 (2)	C7—N2—C4—C5	15.6 (4)
O2—S1—C1—C6	175.7 (2)	C3—C4—C5—C6	0.5 (4)
O1—S1—C1—C6	46.8 (2)	N2—C4—C5—C6	178.6 (2)
N1—S1—C1—C6	−68.2 (2)	C4—C5—C6—C1	−0.1 (4)
C6—C1—C2—C3	−0.3 (4)	C2—C1—C6—C5	0.0 (4)
S1—C1—C2—C3	−177.88 (19)	S1—C1—C6—C5	177.6 (2)
C1—C2—C3—C4	0.7 (3)	C4—N2—C7—O3	0.5 (4)
C2—C3—C4—C5	−0.8 (4)	C4—N2—C7—C8	−177.7 (2)

### Hydrogen-bond geometry (Å, º)

**Table:**

*D*—H···*A*	*D*—H	H···*A*	*D*···*A*	*D*—H···*A*
N1—H1···O3^i^	0.88 (1)	2.08 (1)	2.935 (3)	163 (3)
N1—H2···O3^ii^	0.89 (1)	2.04 (1)	2.929 (3)	178 (3)
N2—H3···O1^iii^	0.88 (1)	2.34 (2)	3.156 (3)	155 (2)

Symmetry codes: (i) −*y*+3/2, −*x*+3/2, *z*+1/2; (ii) −*x*+1, −*y*+2, *z*; (iii) *x*+1/2, −*y*+3/2, −*z*+3/2.
